# Malaria vaccine research and development: the role of the WHO MALVAC committee

**DOI:** 10.1186/1475-2875-12-362

**Published:** 2013-10-10

**Authors:** Geoffrey AT Targett, Vasee S Moorthy, Graham V Brown

**Affiliations:** 1 Faculty of Infectious and Tropical Diseases, London School of Hygiene & Tropical Medicine, Keppel Street, London WC1E 7HT UK; 2 Initiative for Vaccine Research, Department of Immunization, Vaccines and Biologicals, World Health Organization, Avenue Appia 20, 1211-CH 27 Geneva, Switzerland; 3 Nossal Institute for Global Health, University of Melbourne, Carlton, Victoria 3010 Australia

**Keywords:** Malaria, Clinical trials of vaccines, Efficacy measures, Transmission, Modelling, *P*. *vivax* vaccine research

## Abstract

The WHO Malaria Vaccine Advisory Committee (MALVAC) provides advice to WHO on strategic priorities, activities and technical issues related to global efforts to develop vaccines against malaria. MALVAC convened a series of meetings to obtain expert, impartial consensus views on the priorities and best practice for vaccine-related research and development strategies. The technical areas covered during these consultations included: guidance on clinical trial design for candidate sporozoite and asexual blood stage vaccines; measures of efficacy of malaria vaccines in Phase IIb and Phase III trials; standardization of immunoassays; the challenges of developing assays and designing trials for interventions against malaria transmission; modelling impact of anti-malarial interventions; whole organism malaria vaccines, and *Plasmodium vivax* vaccine-related research and evaluation. These informed discussions and opinions are summarized here to provide guidance on harmonization of strategies to help ensure high standards of practice and comparability between centres and the outcome of vaccine trials.

## Background

Much has been achieved in controlling malaria in many endemic areas of the world [1,2] and further progress is possible through universal access to and use of existing malaria preventive, diagnostic and treatment measures, including transmission reduction through *Anopheles* mosquito vector control. Nevertheless, current tools on their own are unlikely to provide elimination in areas of highest transmission, especially in Africa, and new tools are needed. One or more effective vaccines which can be added to available measures could fill that critical gap and could support malaria control. Despite some investment in research on the development of malaria vaccines over the past thirty years [3,4], currently only one candidate vaccine, RTS,S/AS01, has reached the stage of phase III clinical trials, with the prospect of being submitted for consideration of licensure soon [5]; several others have been tested in phase II field trials [6].

Throughout this period of increased vaccine effort, WHO has had an important role in supporting various aspects of basic research projects through the WHO/UNDP/World Bank Special Programme (WHO/TDR) and has also played a major normative role in providing guidance on many aspects of vaccine development. Landmark meetings were held to highlight the need for improved adjuvants, by bringing together representatives of groups involved in developing and testing new potential products, recognizing that malaria antigens were often involved in the first wave of testing novel agents [7]. WHO worked with funding agencies to convene meetings of scientists, regulators and others to review the state of the art of vaccine development, reviews of key ethical issues [8], and maintains an ongoing record of malaria vaccines under development (known as the Rainbow Table) [3]. Perhaps most importantly, WHO has worked with its advisory committees to gather best evidence then provide guidance for vaccine trial designs that ensure that results generated are relevant for establishing subsequent policy recommendations, to assist national programmes in malaria-endemic countries.

WHO’s advisory committees are a key mechanism for identification of research priorities for immunization and for development of consensus-based guidance on clinical development and testing of vaccines. The WHO Malaria Vaccine Advisory Committee (MALVAC) succeeded IMMAL and VDR Committees, and provides advice to WHO on strategic priorities, activities and technical issues related to global efforts to develop vaccines against malaria, with emphasis on the public health needs of developing countries. In the five years 2008–2013 MALVAC has convened eight key meetings of experts, together with several working groups, whose detailed assessments have provided valuable consensus views on priorities and best practice for selected vaccine-related research and development strategies. By providing the leading independent global malaria vaccine forum for funding agencies, sponsors and investigators, WHO has increased collaboration between key malaria vaccine R&D stakeholders. WHO has also improved the comparability of key endpoints by convening technical groups to provide consensus based protocols and Standard Operating Procedures, ensuring extensive consultation amongst stakeholder groups. The technical areas for these consultations have included design and conduct of sporozoite challenge trials, Standard Operating Procedures for malaria microscopy in challenge trials, [9] optimization of clinical challenge trials for asexual blood stage vaccines, measures of efficacy of malaria vaccines in phase IIb and phase III trials, workshops on standardization of malaria vaccine immunoassays, evaluation of assays and trial designs to be applied to interventions against malaria transmission, development of whole organism vaccines for malaria endemic countries, and priorities in research and development of vaccines for *Plasmodium vivax* and their evaluation. Recently, WHO highlighted the need for information-sharing among HIV, TB and malaria vaccine communities and working with NIAID convened a technical forum on heterologous prime-boost immunization across the three diseases [10]. Furthermore the MALVAC committee will have a key role in advising WHO on the updated version of the Malaria Vaccine Technology Roadmap [11].

These informed and impartial opinions and recommendations are summarized here to provide guidance on harmonization of strategies that will help to ensure high standards of practice, and comparability between centres and the outcome of vaccine trials. Reference is made to reports of the meetings (and to selected other relevant publications) that provide the more detailed discussions on which the recommendations are based. The text below summarizes the reports of individual meetings and should not be considered to be the position or policy of the WHO. For outcomes of individual meetings, readers are directed to the individual meeting reports.

There is a distinct WHO advisory mechanism which provides advice on vaccine candidates in advanced development and approaching possible availability for use. This is known as the Joint Technical Expert Group (JTEG) on malaria vaccines, reporting jointly to the WHO Strategic Advisory Group of Experts on Immunization, and to WHO’s Malaria Policy Advisory Committee [12]. MALVAC will continue to provide advice to WHO on the longer term malaria vaccine R&D considerations, with JTEG available to provide evidence reviews for possible policy recommendations when products become sufficiently advanced.

### Consensus-based guidance on clinical trial design

A vital part of the development of candidate malaria vaccines is the careful planning of all phases of clinical trials, always with a view to enhancing comparability between vaccine clinical trials, sites, and alternative development programmes. A second important objective is generation of data to terminate or advance projects appropriately. Phase I trials are used to determine whether candidate vaccines have the required profile of safety and immunogenicity, with Phase IIa trials in malaria designed to provide actionable information on efficacy, safety and immunogenicity including controlled human malaria infection. They are screening trials intended to select candidate vaccines to take forward into field trials, and to select which vaccine formulations to terminate. These proof of concept studies have usually started with adults in non-endemic areas before moving to the target group of children of an endemic area. Phase IIb and III proof of principle field trials require progressively larger numbers of subjects depending on the primary endpoint to be measured and the controls necessary for comparison. Phase III trials are traditionally designed with the primary objective of providing data suitable for regulatory filing. However a focus in malaria vaccines has been to ensure that data are also suitable for evidence-based public health policy recommendations as far as possible. This can avoid the need for additional Phase III trials.

### Challenge trials in malaria-naïve volunteers

#### Sporozoite challenge trials for pre-erythrocytic and blood-stage vaccines

**Figure 1:**
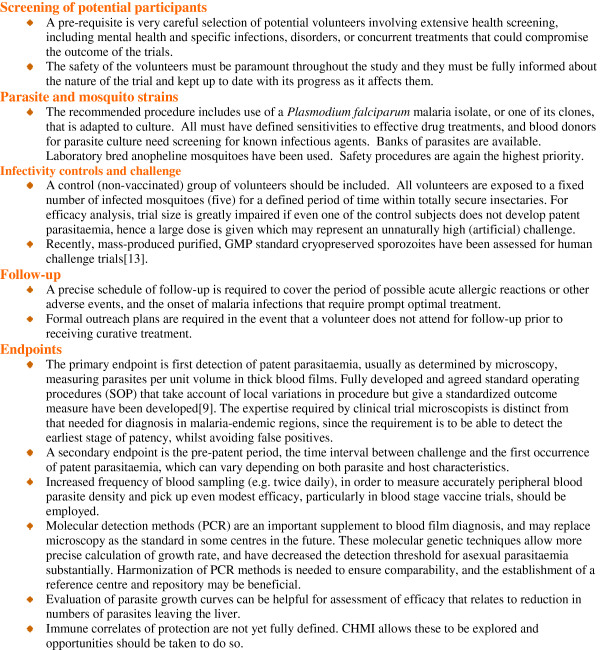
**Standardization and conduct of *P. falciparum *sporozoite controlled human malaria infection trials.** CHMI is used to denote sporozoite challenge but it is also sometimes used too for blood-stage challenge.

Controlled Human Malaria Infections (CHMI) [9,13] are used to assess candidate vaccine efficacy in malaria-naive individuals. Challenge trials in volunteers are important as they inform future clinical trials – whether or not to proceed; dosing, route, schedule and vaccine presentation. This allows iterative improvement of the vaccine construct and its use. Field trials for pre-erythrocytic vaccines should be dependent on first achieving a pre-determined level of efficacy in challenge trials. Future efficacy field trials of new vaccine candidates or combinations may well have to be non-inferiority trials (if RTS,S, for example, is licensed) in regions of reduced transmission, potentially requiring very large sample sizes, thus making them costly and complex with subjects enrolled in several centres. The need to achieve greater confidence in expectations of efficacy prior to initiating multi-million dollar field studies in large numbers of individuals means that even greater reliance may be placed on carefully standardized CHMI studies. They will also be important for the same reasons in go/no go criteria and prioritization of blood stage vaccines (Figure 1). The significant additional challenges with *P. vivax* challenge trials are considered later.

#### Blood stage challenge trials

As an alternative to sporozoites, parasitized erythrocytes can be used to challenge vaccinated individuals [14]. They may have advantages over sporozoite challenge for testing the efficacy of blood-stage vaccines since they allow use of a standardized and low dose challenge and, consequently, a longer period for assessment of induced blood stage immunity before parasites are detected and treatment is required. In contrast, the large sporozoite challenge required to ensure that every control subject is infected may lead to heavy infection and a large numbers of parasites leaving the liver and, as a consequence, earlier detection of parasites requiring treatment.

Rigorous safety depends on having a well-characterized source of erythrocytes free from adventitious agents that meet stringent blood product safety requirements. This is the area of particular concern that for some investigators may still outweigh the perceived benefits of blood-stage challenge. The antigenicity of infected red blood cells in vaccinated volunteers is also a safety issue to be considered. Quantitative PCR monitoring of infection is valuable as it provides a detection threshold of asexual parasitaemia of approximately 20 parasites/ml, well below that possible by microscopy, and potentially increases the time for observations and assessment of efficacy before treatment is required.

There are, however, some drawbacks to use of blood-stage challenge. The challenge bypasses skin and liver stages of infection and removes the possibility of detecting protective cellular immune responses against the late liver stage or antibody against merozoites released from the liver. A low inoculation of parasitized erythrocytes has no counterpart in naturally acquired blood stage infection. Further development of blood stage challenge trials and comparisons with sporozoite challenge are desirable. Development of multiple antigenically distinct parasite strains for evaluation of heterologous protection is also required. Independent evaluation of methods used for modelling parasite growth curves should be made to assist decision-making.

#### Phase IIb and Phase III malaria vaccine trials

Detailed recommendations were made on the implications of different measures of efficacy as they affect vaccine impact, comparability of trials, licensure and wider public health benefits [15]. The conclusions were:

• Field evaluation of a vaccine has to be done in the context of other control measures. Assessing efficacy is complicated for malaria where first infection (or vaccination) gives only partial protection against re-infection and the same individual may have multiple episodes of clinical malaria. The primary measure of efficacy is commonly reported as the incidence of first episode of infection or of clinical malaria. This takes no account of subsequent episodes and, from a public health perspective, reduction in the total number of events in some defined time period following vaccination is more relevant than measuring time to first event as the primary efficacy endpoint.

• Incident malaria infection is a prerequisite of clinical malaria but, additionally, there can be incident infections without clinical symptoms. If protection against incident malaria infection could be used as a correlate for protection against clinical malaria, this would allow smaller trial sizes and less cost (in malarious areas where many trials are likely to be conducted, incidence of infection is high, with the vast majority of susceptible individuals expected to experience infection in a one to three month period at the peak of local transmission).

• A high incidence of malaria and heterogeneity of exposure make estimates of efficacy difficult. In particular, waning of efficacy and heterogeneity in exposure cannot be distinguished by measuring the proportion of individuals remaining disease free at different times after vaccination. Boosting from natural infection should be evaluated. Trial designs in endemic countries will need to be able to detect duration of protection at least up to two years after vaccination, and to rule out deferred or rebound increases in mortality as a consequence of increasing susceptibility following a period of vaccine-induced immunity.

• Evaluation of efficacy in the context of existing malaria preventative interventions is a priority. The dosing schedules required for successful vaccination may have implications for feasibility of scale-up. Possible interference with EPI vaccines in infants should be assessed. The possibility of reduced transmission in some field trial sites will need to be addressed in field trial design; larger enrolments may be required, and because age at risk will have extended beyond the ages for EPI, vaccines may also need to be administered to the older children at risk.

• Safety, immunogenicity and efficacy should be established prior to a vaccine being given to specific high risk groups (e.g. infants, in pregnancy, and immune-compromised individuals), Monitoring and evaluation plans should include both pharmacovigilance and a sustainable disease burden monitoring system. However these pose major challenges in many malaria-endemic settings.

• Impact on malarial transmission (see below) is a key part of assessment of any potential new malaria intervention, although it may not be included in the first Phase III trial. The future combination of single or multi-component candidate malaria vaccines with other malarial interventions should be considered.

• As a general rule methods for design and analysis of Phase III trials should be registered in publicly available data bases before results are unblinded. Data sharing will increase understanding of the likely public health impact of a new vaccine in the context of existing control measures.

• Post-licensure, data should be acquired on long-term effectiveness in multiple transmission settings and changing control measures and epidemiology, noting the consequences when immune individuals have long periods of reduced exposure, for example whether they become more susceptible to the consequences of later episodes of malaria. The long-term safety must also be evaluated.

### Measures of efficacy of interventions against malaria transmission

Reduction in transmission remains the fundamental goal of malaria control and measuring changes in transmission allows a better understanding of the interactions between different interventions (vector control, treatment, vaccination) and their combined impact. Some currently available anti-malarial interventions lead to a reduction in transmission even in highly malarious areas, and transmission reduction is the key metric in a malaria elimination strategy.

Better measures of transmission are needed but, as yet, there are no agreed and standardized measures of malaria transmission and these may need to be different in areas of high and low transmission. Measurement of transmission is costly and time-consuming, requiring repeated observations. In some very low transmission areas, incident infection leads to disease and may, therefore, be a surrogate for ongoing transmission. However, in other areas of very low transmission, substantial numbers of asymptomatic infections have been detected with sensitive molecular tools. At low transmission intensity (for example in some of the environments in which transmission blocking vaccines are likely to be tested and introduced) estimates have very large confidence intervals and surrogates are required (see below). Transmission blocking vaccines (TBV) specifically target sexual or sporogonic stage parasites, or mosquito midgut antigens. However, the so-called vaccines that interrupt malaria transmission (VIMT) may have their primary effect on other of the three main life-cycle stages but, additionally, may have a significant indirect effect on transmission.

The key effect required for sexual stage or mosquito antigen targets is substantial reduction in the proportion of infected mosquitoes. By contrast, the key effect required for pre-erythrocytic vaccines when viewed as VIMT, is a major reduction in the proportion of humans carrying sexual stages.

**Figure 2:**
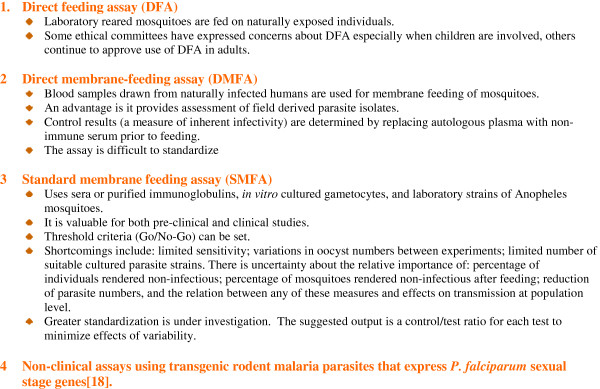
**Assays for evaluation of reduction in transmission from humans to mosquitoes.** A priority is for assays with acceptable precision to improve comparability between trials and to assist regulatory authorities.

A WHO MALVAC meeting considered measures of efficacy of anti-malarial interventions against malaria transmission [16]. The objectives were to evaluate current methods for measurement of malaria transmission and assessment of assays and clinical trial designs that should be applied to measurement of reduction of transmission (of *Plasmodium falciparum*). The measures considered necessary included:

(i) Epidemiological (incidence of new infections)

(ii) Standardized assays of transmission from humans to mosquitoes (Figure 2) [16,17]

(iii) Entomological (estimating new human infections by mosquito measures)

(iv) Surrogate serological and molecular measures, *e.g*. sero-negativity in young children as transmission declines.

A major gap in knowledge is the lack of understanding of the relationship between a result in a trial in a group of vaccinated individuals (each one having a different percentage reduction in oocysts in a different percentage of mosquitoes fed on that person), and the potential effect on transmission. It may be that the most important figure is the proportion of vaccinated individuals who are rendered unable to be a source for feeding mosquitoes to become infectious; another parameter may be the duration of infectiousness of vaccinees.

### Heterogeneity in malaria transmission and focal transmission (hotspots)

There is spatial and temporal heterogeneity in malaria transmission in both low and high transmission endemic areas, seen particularly in incidence of infection in infants and in population based sero-positivity rates. High-density carriers of gametocytes transmit more often in feeding experiments but, importantly, sub-microscopic gametocyte carriers may account for a substantial proportion of transmission. Molecular techniques are required for detection of sub-patent infections as microscopy and rapid diagnostic tests (RDT) miss many asexual blood stage parasites and gametocytes. Targeting young children alone with transmission-reducing interventions is unlikely to be successful, as older children and adults also infect mosquitoes.

### Entomological measures of transmission

The two key measures used to evaluate the impact of interventions are the entomological inoculation rate (EIR), the number of infectious bites per person per unit of time (day, year), and the daily rate of potential transmission by a mosquito population known as the vectorial capacity. Human landing catches (HLC) are used as the standard for mosquito collection but subjects vary in their attractiveness and there are ethical concerns. Light traps are used as an alternative and to compare indoor/outdoor biting and time of biting. They lack reliability outdoors and the mosquito composition may vary from HLC.

The minimum EIR measureable reliably is 5–10 and transmission-blocking vaccine trials are likely to be conducted at field sites with low to moderate EIR. Non-random distribution of vectors, as well as endophilic or exophilic behaviour, present problems. Serological measures of exposure of humans to *Anopheles* salivary peptides can be used to measure changes such as the impact of ITNs on biting. ELISA measures of sporozoite infections in mosquitoes are convenient but some reports have indicated a high level of false positivity which gives an overestimate of infected mosquito rates. Measuring the infection rate according to age in wild caught mosquitoes requires labour intensive age grading and large numbers of mosquitoes.

### Clinical trials of transmission-blocking vaccines

#### Phase Ia, Ib and IIb vaccine studies

Safety and immunogenicity and functional activity will be required, as already indicated, before progression from Phase I in naïve volunteers to endemic country trials. Intermediate proof of principle may be shown by demonstrating antibody-mediated reduction in infectivity of humans to mosquitoes in membrane-feeding assays. Efforts to validate or quantify the assays are required and, currently, Go/No-go criteria from Phase I to Phase IIb trials are not agreed. The possibility of demonstrating a reduction in infectivity of vaccinated humans to mosquitoes in Phase IIb also needs further standardization of study design and analysis.

#### Phase III design for a malaria TBV

Field trial sites for phase III trials need to be characterized in terms of parasite prevalence and heterogeneity (hotspots), infectious reservoirs, incidence of infection or clinical malaria in infants; mosquito exposure; serology and natural transmission-blocking immunity. There are challenges for design of clinical trials in both high and low transmission sites as an effect may be difficult to show in one case, and power may be lacking to show an effect in the other. Furthermore, transmission may change over the several years of the study. Trial design will be complex. Cluster-randomized efficacy trials may be required, and a step-wedge design has also been suggested.

It is important to distinguish between those interventions with expected direct effects on other stages of the life cycle and possible indirect effects on transmission (VIMT), and interventions only expected to have effects on transmission (TBV). Cluster randomized trials can be used for any VIMT, measuring both the direct and indirect effects, as well as for TBV. Contamination amongst clusters is a problem and a 2-4 km buffer zone was recommended during a MALVAC consultation.

Surrogate endpoints for assessing transmission-blocking vaccines in humans were proposed [16] using *e g.* oocyst prevalence in membrane feeding. Whether conducted pre- or post-licensure (i.e. Phase III or Phase IV), trials designed to evaluate the effect of a TBV on transmission will be necessary and could incorporate the surrogates or biomarkers as additional endpoints. The primary endpoint for a Phase III trial of a TBV would be incidence of human infection, and sensitive assays for detection of infection are necessary for such a purpose. Secondary endpoints may include incidence of clinical cases and entomological measures such as EIR, although it should be noted that in trial settings, transmission might be close to the lowest transmission level at which EIR measurements are reliable. Tertiary endpoints derived from serological or nucleic assay measures become important for surveillance–related operational research.

Age groups should always include children through to adolescents, but further research and modelling on the distribution of the infectious reservoir will be required to determine whether this needs to be extended to young adults or even the whole population (except groups who could be at high risk of vaccine side-effects). A follow-up of at least two years is required.

### Transmission-blocking vaccine development

Good progress has been made using different approaches to development of transmission-blocking vaccines that target sexual or sporogonic stages of the life cycle. Candidate vaccines expressing antigens of *P. falciparum* involved in fertilization induced strong transmission-blocking activity assessed with standard membrane feeding assays [18]. Various vaccine formulations of a post-fertilization molecule had equally high transmission-blocking activity [19,20]. Conjugates have been developed for phase I trials and formulation of vaccines to further enhance their immunogenicity is under investigation. Combination vaccines with pre-erythrocytic and sexual stage components are planned. Preferred product characteristics require further work.

### Modelling impact of anti-malarial interventions

Leading modelling groups combined to present opinions on the questions relating to impact of interventions that are best answered by modelling, the outcomes of particular interest to show impact on transmission, and what field data are necessary to make models more robust [16]. It was strongly recommended that field researchers work with modelling groups to improve outputs as the models need more information on:

• different levels and heterogeneity of transmission;

• the influence of superinfection on duration of infection and infectivity;

• the infectious reservoir and age structure in low transmission settings;

• the rate of acquisition of immunity in different transmission settings and loss of immunity when transmission is reduced;

• vector species and densities;

• the relationship between transmission and climate;

• human behaviour and socio-economic factors.

There is a large discrepancy between estimates of R_o_ in high transmission settings measured by the number of infections per person per unit of time-the force of infection (FOI), or by the entomological inoculation rate (EIR). The effect of a TBV can be measured by the proportionate reduction in probability that a mosquito acquires infection at a given feed. Prevalence of infection in mosquitoes is probably more important than density of infection.

Specific TBV may not be better at interrupting transmission than other vaccines, all of which could have the additional benefit of an indirect effect on transmission. However, chances of elimination depend on coverage, initial transmission level, good surveillance, natural immunity and human population size. Vaccines need consideration in terms of both transient dynamics and effects over time. Transmission is heterogeneous with respect to intensity, seasonality, vector species and foci of infections. Modelling should be able to accommodate this heterogeneity together with human infectiousness and immunity in order to inform discussions about who and when to vaccinate with a transmission-blocking vaccine and which other interventions should be included [21]. Sensitivity analyses are important to provide information on key parameters driving uncertainty in the models. Further transmission-related epidemiological studies may be very helpful for data fitting, depending on the study design.

### Whole organism malaria vaccines

#### Attenuated sporozoite vaccines

Humans exposed to bites of large numbers of infected irradiated mosquitoes have shown a high level of short-term protection in controlled human malaria infection trials [22-25]. This has provided a basis for research into the development of attenuated sporozoite vaccines. An irradiated sporozoite production programme using GMP procedures has been developed and has made available clinical grade materials for phase I/IIa vaccine trials [26]. Proof of concept studies have begun in adults with radiation-attenuated vaccines [27].

Considerations that require evaluation and further research include the size of the vaccine dose, the number of doses and their timing, routes of administration, longevity of protection, boosting, immune correlates of protection, protection against heterologous challenge, and storage, stability and transport of the vaccines. Genetically-attenuated sporozoites potentially provide an alternative to irradiated sporozoites as vaccines but the possibility of reversion to virulence or under-attenuation with both irradiated and genetically attenuated sporozoites requires careful assessment [28].

#### Sporozoite inoculation and chemoprophylaxis

Volunteers exposed to bites of mosquitoes infected with *P. falciparum* while simultaneously given chloroquine prophylaxis were fully protected against a challenge infection given 2.5 years later opening a new approach to studies on mechanisms of pre-erythrocytic protective immunity [29].

#### Asexual blood stage whole organism vaccines

Low dose parasitized erythrocyte inoculation, leading to infection controlled by chemotherapy, can provide protection against challenge infections in malaria naive volunteers without induction of detectable antibody [22,30]. A key safety requirement is a constant source of erythrocytes and other blood products free from adventitious agents. The antigenicity of infected red blood cells in vaccinated volunteers needs to be ascertained but has not been a problem so far. This system of “experimental medicine” can provide insights into protective immune responses induced, and antigenic correlates of protection to guide later production of defined antigen vaccines, even if the concept does not progress as a candidate vaccine in its own right. Issues identified for further studies include whether antibody in malaria-exposed individuals would interfere with the vaccine and whether adjuvants are required to help promote the desired immune response.

### Plasmodium vivax *vaccine-related research*

Much of the vaccine-related research and planning is focussed on *P. falciparum* malaria yet the burden of disease due to *P. vivax* is high. The global population at risk of *P. vivax* has been estimated at over 2 billion [31]. Outside Africa, *P. vivax* accounts for more than 50 per cent of malaria cases. There are wide uncertainty ranges around global disease burden estimates for *P. vivax*, although it is agreed that *P. vivax* represents a major cause of disease in Asia and South America in particular. There is increasing evidence for *P. vivax* infection in more settings in Africa [32].

Two WHO MALVAC meetings considered priorities in research and development of vaccines against *P. vivax*[33] and provided detailed guidance on the evaluation of *P. vivax* vaccines in naturally exposed populations [34].

#### Research required for development of vaccines against vivax malaria

*Plasmodium vivax* has aspects of its biology and life cycle that are species-specific when compared with *P. falciparum* and these make all control measures more difficult: infectious gametocytes appear early in the infection; development in the liver includes formation of persisting hypnozoites; the extrinsic cycle is shorter and is completed at a lower temperature; there is efficient transmission even when mosquitoes are highly seasonal. *Plasmodium vivax* and *P. falciparum* are commonly sympatric, particularly where transmission of *P. vivax* is high. There is conflicting evidence on the impact of one species on the other.

Detailed baseline studies that are required include: burden of disease; the clinical spectrum and incidence of severe malaria and mortality; relapse patterns; strain heterogeneity; interactions with other species and seasonality of *P. vivax* and *P. falciparum*; immunology specific to *P. vivax* and in mixed infections; economic analyses. These will guide determination of sample size in trials, timing of vaccination, and surveys in relation to transmission.

Data are required particularly on sensitivity to drugs needed for radical treatment, especially to primaquine, and on the rate of G6PD deficiency. No radical cure treatment is fully effective. Though pre-erythrocytic and asexual blood-stage vaccines are needed, special consideration of the role of vaccines in blocking transmission is required. In the long-term quest for eradication, the MalERA consultation [35] acknowledged that further basic research to achieve this goal must include as priorities continuous *in vitro* culture of asexual blood stage forms and gametocytes, and hypnozoite production in relevant cell lines. Non-human models of *P. vivax* are available, but these match human infections in variable ways and should, therefore, be chosen according to the questions being asked.

### Clinical trials

*Plasmodium vivax* presents some substantial additional challenges when compared with *P. falciparum;* these are highlighted below. It is noted that testing for protection against hypnozoites is currently not possible.

#### Controlled human malaria infection trials

Like *P. falciparum*, a standardized human sporozoite challenge model is required for evaluation of pre-erythrocytic vaccine candidates. However, it is not yet possible to challenge with the same highly characterized clone of *P. vivax* due to the lack of continuous asexual and gametocyte *in vitro* culture systems. Challenges have been conducted with varying numbers (3–9) of mosquitoes infected with different *P. vivax* strains. Challenge studies thus include the additional issues of challenge with wild type parasites and the possibility of relapses. There are plans to produce vialled, cryopreserved *P. vivax* sporozoites for a needle-based *P. vivax* challenge model (cf *P. falciparum*). As with *P. falciparum*, Phase IIa trials are helpful for pre-erythrocytic vaccines, but somewhat contentious for blood-stage vaccine candidates.

#### *Phase IIb and Phase III trial designs for* Plasmodium vivax

The sequence of trials required is similar to that for *P. falciparum.* With *P. vivax*, vaccine profiles are based on the need to identify the target groups:

(i) to prevent infection in all age groups in low endemic areas. Mass vaccination may be aimed at elimination or outbreak control.

(ii) to prevent disease in infants and children in high transmission areas – where *P. vivax and P. falciparum* infections are commonly co-endemic.

(iii) to vaccinate migrants, who need long-term protection to cover relapses.

Initial Phase IIb/III trials should be in highly endemic areas (in children under five years of age). Later trials need to include additional target groups, according to the endemicity of infection. Major challenges to study design and sample size calculations include individuals who have asymptomatic parasitaemia, hypnozoite infections, and *P. falciparum* co-infections that require treatment. Virtually nothing is known about immunity to hypnozoites and assessment of their presence is currently not possible; meaning that production of a highly desirable therapeutic vaccine is so far out of reach.

For pre-erythrocytic vaccines, a treatment-reinfection design (involving radical treatment to remove hypnozoites) is best if the primary endpoint is incidence of infections. It is difficult to detect mixed infections without molecular techniques. Radical cure to eliminate hypnozoites does however present problems (e.g. haemolytic effects, compliance, and not being fully effective). For blood stage vaccines radical cure is not required if the vaccine will protect equally well against relapse infections.

### Plasmodium vivax *efficacy endpoints and follow-up*

It is proposed that, for first pre-erythrocytic vaccine trials, incidence of infection is the primary endpoint required. For subsequent trials, incidence of disease should be determined. For blood stage vaccines, the primary efficacy endpoint needed is the incidence of uncomplicated *P. vivax.*

Particular complications of assessment of *P. vivax* trials and follow-up include:

• Genetic diversity of isolates

• Co-infection with *P. falciparum* and the need to treat this infection if it becomes symptomatic

• Assessment of efficacy against relapse infections and the consequent need for long-term follow-up

## Conclusions

WHO has played a major convening role in the past three decades in establishing a normative framework for critical aspects of vaccine development as antigenically-defined subunit vaccines, novel platforms and new adjuvants were introduced for human use, often for the first time in association with malaria vaccine studies. The importance of early and close involvement of scientists, developers, regulators, and public health physicians has enabled continuing close cooperation in later stages as developers evaluate vaccines in ways which will enable technical advisory bodies of WHO to have access to the data that will enable them to provide guidance for country programmes.

This review has focussed on the contributions to clinical trial design. In many cases, the discipline has moved from theory to practice, with large numbers of trials of pre-erythrocytic and asexual stage antigens with different adjuvants now completed and published, so that new protocols can benefit from practical experience added to theoretical considerations. The context has also changed with many trial sites having experienced gratifying reduction in morbidity and mortality, one consequence being the requirements for larger multi-centre studies and complications of interpretation from sites with different intensities of transmission. The reduced transmission has also caused many to focus mainly on the possible contribution of vaccines directed against any stages to reduction of transmission and possible elimination of malaria. Non-falciparum species have taken a backseat role at this stage and ongoing debate continues about the best way of reporting efficacy data. As much is now known and published on pre-erythrocytic and asexual stage vaccine trials, at least to proof of concept stage (probably better described as experimental medicine rather than product development), the review has given greater attention to what lies ahead. The focus is on design of trials for which there is little or no experience, namely trials of vaccines for *P. vivax*, and trials for assessment of reduction of transmission, and for which preferred product characteristics are still the subject of debate (for example with respect to efficacy against hypnozoites or go/no go criteria for transmission blocking studies).

The field is forever changing, with falling transmission leading to changed populations at risk, including older children beyond the age of the EPI schedule or migrants at risk of epidemic malaria. Vaccines that could be used locally for control or elimination of multi-drug resistant malaria require consideration of new product profiles and novel ways of assessing combined efforts with multiple tools for malaria control that call for better ways of measuring effects when transmission is low and non-uniformly distributed.

The review has highlighted the past and ongoing contribution made by WHO in convening groups to address key issues for investigators, vaccine developers, regulatory authorities and funders in ensuring the most efficient use of resources for developing much-needed vaccines for use in malaria endemic countries.

## Competing interests

The authors declare that they have no competing interests.

## Authors’ contributions

This integration of discussions and recommendations from MALVAC meetings was suggested by VM. The draft was written by GT. All three authors contributed to its revision and approved the final manuscript.
